# High sleep fragmentation parallels poor subjective sleep quality during the third wave of the Covid‐19 pandemic: An actigraphic study

**DOI:** 10.1111/jsr.13519

**Published:** 2021-11-19

**Authors:** Francesca Conte, Oreste De Rosa, Marissa Lynn Rescott, Teresa Pia Arabia, Paolo D’Onofrio, Alessio Lustro, Serena Malloggi, Danila Molinaro, Paola Spagnoli, Fiorenza Giganti, Giuseppe Barbato, Gianluca Ficca

**Affiliations:** ^1^ Department of Psychology University of Campania L. Vanvitelli Caserta Italy; ^2^ Department of Psychology University of Stockholm Stockholm Sweden; ^3^ Department NEUROFARBA University of Firenze Firenze Italy

**Keywords:** actigraphy, Covid‐19 pandemic, objective sleep quality, sleep schedules, subjective sleep quality

## Abstract

Studies on sleep during the Covid‐19 pandemic have mostly been conducted during the first wave of contagion (spring 2020). To follow up on two Italian studies addressing subjective sleep features during the second wave (autumn 2020), here we assess sleep during the third wave (spring 2021) in a sample of healthy adults from Campania (Southern Italy). Actigraphic data (on 2 nights) and the Pittsburgh Sleep Quality Index were collected from 82 participants (40 F, mean age: 32.5 ± 11.5 years) from 11 March to 18 April 2021, when Campania was classified as a “red zone”, i.e. it was subjected to strict restrictions, only slightly looser than those characterizing the first national lockdown (spring 2020). Although objective sleep duration and architecture appeared in the normal range, the presence of disrupted sleep was indexed by a relevant degree of sleep fragmentation (number of awakenings ≥ 1 min: 12.7 ± 6.12; number of awakenings ≥ 5 min: 3.04 ± 1.52), paralleled by poor subjective sleep quality (Pittsburgh Sleep Quality Index global score: 5.77 ± 2.58). These data suggest that the relevant subjective sleep impairments reported during the first wave could have relied on subtle sleep disruptions that were undetected by the few objective sleep studies from the same period. Taken together with sleep data on previous phases of the pandemic, our findings show that the detrimental effects on sleep determined by the initial pandemic outbreak have not abated across the subsequent waves of contagion, and highlight the need for interventions addressing sleep health in global emergencies.

## 1 INTRODUCTION

Early evidence from the Covid‐19 crisis has shown wide‐ranging disruptions to personal schedules, psychological health and sleep throughout the world, with pooled data from international populations placing the prevalence of sleep problems at 35.7% (for a review, see Jahrami et al., 2021).

During the first lockdown in Italy, individuals reported delayed sleep schedules, increased time in bed (TIB) and poorer sleep quality compared with before the lockdown (Casagrande, Favieri, Tambelli, & Forte, 2020; Cellini, Canale, Mioni, & Costa, 2020; Cellini et al., 2021; Gualano, Lo Moro, Voglino, Bert, & Siliquini, 2020). Over 40% of an Italian sample reported sleep disturbances (Gualano et al., 2020) and 18% met criteria for a diagnosis of clinical insomnia (Bacaro et al., 2020). Taken alongside results from surveys conducted worldwide, it appears that there has been a global decline in sleep quality (Huang & Zhao, 2020; Kokou‐Kpolou, Megalakaki, Laimou, & Kousouri, 2020; Leone, Sigman, & Golombek, 2020; Stanton et al., 2020; Voitsidis et al., 2020).

Two Italian surveys, conducted longitudinally across the first and second pandemic lockdowns (spring and autumn 2020, respectively), show that the impoverishment of sleep quality persisted through the waves of contagion (Conte, Cellini, et al., 2021; Salfi, D'Atri, Tempesta, & Ferrara, 2021). In Italy, in fact, the loosening of restrictions over the summer 2020 resulted in a second, larger wave of infections, to the point that another lockdown, though slightly less restrictive, was mandated in November 2020. Despite the effectiveness of these new measures, a third wave of contagion occurred toward the end of winter, so that most Italian regions underwent a third lockdown in March 2021.

Here we assess objective and subjective sleep features through actigraphic recordings and the Pittsburgh Sleep Quality Index (PSQI; Buysse, Reynolds, Monk, Berman, & Kupfer, 1989), respectively, during the third Italian lockdown in a sample of healthy adults, in order to describe the longitudinal evolution of the pandemic's effects on sleep schedules and quality.

An additional aim is to specifically assess sleep fragmentation, which has been neglected in the few objective sleep studies from the first pandemic wave (Ong et al., 2021; Pépin et al., 2021; Sañudo, Fennell, & Sánchez‐Oliver, 2020; Wang, He, Gao, Gao, & Lei, 2021). Indeed, these studies point to a milder impact of the pandemic on objective sleep quality than that suggested by the survey studies reviewed above: for instance, no changes in sleep efficiency (SE) or wake after sleep onset (WASO) were found during the lockdowns (Ong et al., 2021; Pépin et al., 2021). Therefore, a more in‐depth evaluation of sleep fragmentation measures, consistently reported as main determinants of perceived sleep quality (Conte, Cerasuolo, et al., 2021; Della Monica, Johnsen, Atzori, Groeger, & Dijk, 2018), could shed light on the discrepancy between subjective and objective assessments of sleep during the pandemic.

Finally, we also address gender differences in subjective and objective sleep measures, in order to compare our findings with data collected during previous waves of the pandemic, which point to female gender as a risk factor for greater worsening of sleep quality with the Covid‐19 emergency (Casagrande et al., 2020; Cellini et al., 2021).

## 2 MATERIALS AND METHODS

### 2.1 Participants and procedure

The data collection phase was conducted from 11 March to 18 April 2021, i.e. when Campania, along with most other Italian regions, was considered a “red zone” according to the Governmental Decree of 3 November 2020. Since this decree, Italian regions are being classified as red, orange, yellow or white zones on a weekly basis, based on a set of risk parameters including the number of Covid‐19 cases per inhabitant. “Red zones” are the areas considered at highest risk of contagion spread and thus subjected to the greatest restrictions: movements outside of home are not allowed except for basic necessities (related to work, health, grocery shopping, assistance), with the requirement to carry documentation of essential travel at all times; moving across municipalities is prohibited unless there are exceptional work‐ or health‐related reasons. Only essential shops (such as pharmacies) are allowed to be open. Bars' and restaurants' services are limited to takeaway (until 22:00 hours) and home delivery. Cinemas, theatres, museums and gyms are also closed. All in‐presence activities of schools, universities and team sports are suspended; religious services may continue in strict accordance to social distancing norms.

This set of restrictions is very similar to that adopted during the first, national lockdown, which lasted throughout spring 2020, with the main difference being that limitations were somewhat more relaxed during the November and March “red zone” periods: a higher number of work activities requiring physical presence were possible, police controls were less strict and a few public events (such as some religious services) were allowed to be organized with social distancing precautions. The fact that most Italian regions showed a similar trend since November 2020 (with the implementation of “red zone” limitations for about a month in November–December 2020 and again in March–April 2021) allows to clearly identify, in Italy, a second and third wave of contagion, based both on number of Covid‐19 cases and on severity of restrictions, and to compare sleep data across the waves.

The recruitment phase was conducted along with data collection throughout the “red zone” period (11 March to 18 April), and was ended as soon as the loosening of restrictions was announced (i.e. Campania becoming an “orange zone”), in order to assure that all participants were evaluated under the same conditions. Specifically, a convenience sample of 87 volunteers from the metropolitan areas of Caserta and Napoli (Campania region, Italy) was screened through a brief telephone interview, to collect general demographic data and information on medical conditions and health habits (including specific questions on somatic and psychiatric disorders, on sleep disorder symptoms and on substance use). Inclusion criteria were: age 18–60 years; absence of any somatic or psychiatric illness; absence of any sleep apnea, respiratory or movement disorder symptoms; having a regular sleep/wake cycle; no history of drug or alcohol abuse; limited consumption of caffeine (no more than 150 mg caffeine per day, corresponding to about three cups of espresso or one cup of American coffee) and alcohol (no more than 250 ml per day, i.e. about a pint of standard beer, a full glass of wine, or a small liquor shot). Five volunteers had to be excluded because of: sleep apnea symptoms (1 subject), anxiety symptoms (2 subjects), regular consumption of caffeine and/or alcohol exceeding the criterion limit (2 subjects). The final sample consisted of 82 participants (40 F, 48.78%; 42 M, 51.22%; age range: 18–56 years; mean age: 32.5 ± 11.5 years).

All participants signed an informed consent prior to participation, and received no money or credit compensation for their participation.

Each subject wore an actigraph on his non‐dominant wrist for about 40 hr (on weekdays only): the actigraph was delivered in the afternoon and retrieved the morning after the second recording night. Participants were also requested to fill in the Italian version of the PSQI (Curcio et al., 2013), as well as two sleep diaries (upon awakening on the morning after each night of recording), and to maintain their regular sleep/wake habits during recording days.

The Ethical Committee of the Department of Psychology, University of Campania “Vanvitelli” approved the research protocol (code 15/2021), and certified that the involvement of human participants was performed according to acceptable standards. All methods were carried out in accordance with relevant guidelines and regulations.

### 2.2 Actigraphic sleep analysis

The actigraphs were Motionlogger^®^ Microwatches (Ambulatory Monitoring). The analysis of sleep data was performed on the two night periods, with a 30‐s epoch time scale, by means of the Action‐W 2 software, which uses the Cole–Kripke algorithm (Cole, Kripke, Gruen, Mullaney, & Gillin, 1992) to extract sleep variables. The resting period (i.e. lights off/on times) was automatically defined by the Action‐W 2 software. Specifically, the variables we extracted were: bedtime (i.e. time at which the subject goes to bed), rise time (i.e. time at which the subject rises from bed), sleep midpoint (i.e. midpoint between the first and last epoch scored as sleep), TIB (i.e. total amount of time from bedtime to rise time), total sleep time (TST; i.e. total amount of time spent in sleep), sleep‐onset latency (SOL; i.e. amount of time from bedtime to the first epoch scored as sleep), WASO (i.e. total duration of wake between sleep onset and wake time), SE (i.e. 100* TST/TIB), number of awakenings lasting ≥ 1 min (i.e. number of blocks of at least 2 contiguous wake epochs), mean duration of awakenings, number of long awakenings (lasting ≥ 5 min), duration of longest awakening.

Further, from these automatically extracted variables we calculated: wake time (i.e. time of morning final awakening), sleep period time (SPT; i.e. total amount of time from the first epoch scored as sleep to wake time), WASO percentage (WASO%, i.e. percentage of WASO over SPT), frequency of awakenings lasting ≥ 1 min hr^−1^ of TST, frequency of long awakenings (lasting ≥ 5 min) per hour of TST.

### 2.3 Data analysis

Descriptive statistics are reported as mean ± standard deviation.

In order to be able to pool data from the 2 nights of recording, we checked that actigraphic parameters did not significantly differ between the 2 nights. This was done using Student's *t*‐test for sleep schedule variables (bedtime, wake time, rise time and sleep midpoint) and Mann–Whitney's test for all other objective sleep parameters, which were not normally distributed (as assessed through the Shapiro–Wilk test).

Descriptive data on actigraphic variables are reported as the average between the 2 nights of recording. Similarly, analyses of gender differences in actigraphic parameters were conducted on values averaged between the 2 nights.

Gender differences in age and sleep schedule variables were analysed through Student's *t*‐test, whereas those in PSQI scores, objective sleep architecture and objective sleep fragmentation variables were evaluated through the Mann–Whitney test due to non‐normal distribution.

Furthermore, to assess possible effects of Daylight Saving Time (DST; introduced on 28 March), we analysed differences in actigraphic variables (averaged between the 2 nights) between subjects who participated in the study before that date (*n* = 63; 34 F, 29 M; mean age: 34.5 ± 11.7 years) and those who participated afterwards (*n* = 19; 6 F, 13 M; mean age: 25.7 ± 7.72 years). Sleep schedule measures were assessed through Student's *t*‐test, while sleep architecture and fragmentation variables were analysed by means of the Mann–Whitney test.

Cohen's *d* and 95% confidence intervals are reported for parametric statistics and rank biserial correlations for non‐parametric tests.

All analyses were performed by means of JAMOVI 1.6.16 (The Jamovi Project); significance was set at *p* ≤ 0.05.

## 3 RESULTS

### 3.1 Subjective sleep quality

The average PSQI global score was 5.77 ± 2.58, indicating a mild degree of poor subjective sleep quality. Specifically, 46.34% (*n* = 38) of subjects were classified as good sleepers (PSQI score ≤ 5; Buysse et al., 1989), and the remaining 53.66% (*n* = 44) as poor sleepers (PSQI score > 5; Buysse et al., 1989). Men and women are equally distributed between the two groups (good sleepers: 18 F, 20 M; poor sleepers: 22 F, 22 M). Scores at the PSQI subscales (range 0–3 for each subscale; Buysse et al., 1989) are reported in Table 1.

**TABLE 1:** Scores at PSQI subscales

PSQI subscales (m ± SD)	
Sleep quality	1.24 ± 0.65
Sleep latency	1.17 ± 0.87
Sleep duration	0.65 ± 0.65
Sleep efficiency	0.73 ± 1.01
Sleep disturbances	1.16 ± 0.48
Use of sleep medications	0.04 ± 0.34
Daytime dysfunction	0.82 ± 1.16

Higher scores indicate worse sleep quality, longer sleep latency, shorter sleep duration, lower sleep efficiency, greater sleep disturbances, greater use of sleep medications, greater daytime dysfunction, respectively (Buysse et al., 1989).

### 3.2 Objective sleep quality

No significant differences were found in any actigraphic sleep parameter between the 2 nights of recording.

**FIGURE 1:**
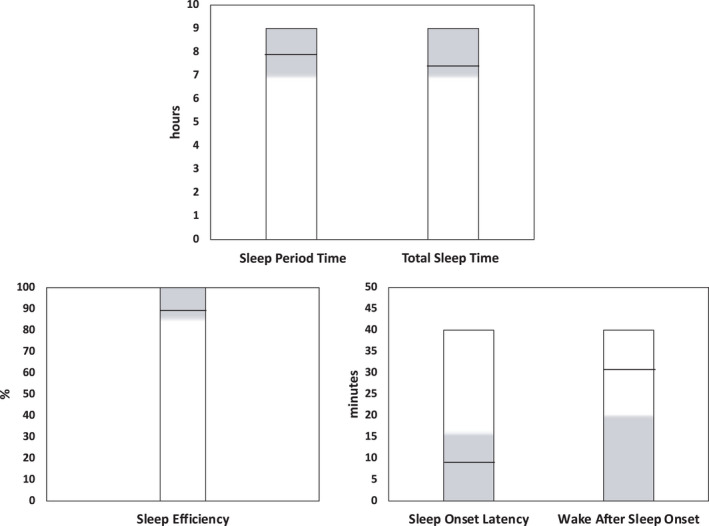
Objective sleep architecture parameters in our participants, in reference to values recommended by the National Sleep Foundation (NSF) for each parameter. Grey areas represent recommended ranges for sleep duration (Hirshkowitz et al., 2015), sleep efficiency (SE), sleep‐onset latency (SOL) and wake after sleep onset (WASO; Ohayon et al., 2016). Black lines indicate the average value for each parameter observed in our sample

Descriptive data on sleep schedules and sleep fragmentation are reported in Table 2, whereas Figure 1 displays sleep architecture variables, in reference to the values recommended for each parameter by the National Sleep Foundation (NSF; Hirshkowitz et al., 2015; Ohayon et al., 2016). TIB and WASO%, not shown in the figure, are 8.09 ± 1.10 hr and 6.71% ± 5.82%, respectively.

**TABLE 2:** Actigraphic data on sleep schedules and sleep fragmentation

Sleep Schedules (m ± SD)	
Bedtime (hr:min)	00:33 ± 1:36
Wake time (hr:min)	08:33 ± 1:22
Rise time (hr:min)	08:41 ± 1:19
Sleep midpoint (hr:min)	04:36 ± 1:21

Sleep Fragmentation (m ± SD)	
Number of awakenings ≥ 1 min	12.7 ± 6.12
Mean duration of awakenings ≥ 1 min (min)	4.09 ± 2.72
Frequency of awakenings ≥ 1 min/TSThr	1.78 ± 0.94
Number of long awakenings (≥ 5 min)	3.04 ± 1.52
Duration of longest awakening (min)	15.6 ± 9.22
Frequency of long awakenings (≥ 5 min)/TSThr	0.46 ± 0.43

TST, total sleep time.

### 3.3 Gender differences

Males and females did not differ in age (M: 32.7 ± 10.7 versus F: 32.3 ± 12.4, Student's *t* = 0.163, *p* = 0.871, Cohen's *d* = 0.036, 95%CI− = −0.40, 95%CI+ = 0.47), nor did gender differences emerge in PSQI global score or in any PSQI subscale (Table 3). Instead, men and women differed in several objective sleep parameters (Table 4), with men showing overall lower sleep quality as indexed by several variables.

**TABLE 3:** Gender differences in PSQI global score and sub‐scores

PSQI subscales	Gender	m ± SD	Mann–Whitney's *U*	*p*	Effect size
Sleep quality	M	1.19 ± 0.63	790	0.602	0.059
	F	1.30 ± 0.68			
Sleep latency	M	1.14 ± 0.89	802	0.713	0.045
	F	1.20 ± 0.85			
Sleep duration	M	0.66 ± 0.68	840	10.000	0.000
	F	0.65 ± 0.62			
Sleep efficiency	M	0.57 ± 0.88	712	0.183	0.152
	F	0.90 ± 1.10			
Sleep disturbances	M	1.21 ± 0.47	756	0.306	0.100
	F	1.10 ± 0.49			
Use of sleep medication	M	0.00 ± 0.00	798	0.150	0.050
	F	0.10 ± 0.49			
Daytime dysfunction	M	0.73 ± 0.73	831	0.927	0.013
	F	0.92 ± 1.49			
PSQI global score	M	5.61 ± 2.54	814	0.812	0.031
	F	5.92 ± 2.63			

Higher scores indicate worse sleep quality, longer sleep latency, shorter sleep duration, lower sleep efficiency, greater sleep disturbances, greater use of sleep medications, and greater daytime dysfunction, respectively (Buysse et al., 1989).

PSQI, Pittsburgh Sleep Quality Index.

**TABLE 4:** Gender differences in objective sleep measures

Sleep schedules	Gender	m ± SD	Student's *t*	*p*	Effect size	95%CI−	95%CI+
Bedtime (hr:min)	M	01:00 ± 1:45	2.76	**0.007**	0.728	0.15	1.37
	F	00:04 ± 1:17					
Wake time (hr:min)	M	08:38 ± 1:22	0.577	0.565	0.128	−0.25	0.46
	F	08:28 ± 1:22					
Rise time (hr:min)	M	08:78 ± 1:21	0.596	0.553	0.132	−0.24	0.45
	F	08:60 ± 1:19					
Sleep midpoint (hr:min)	M	04:53 ± 1:28	1.945	0.055	0.430	−0.00	1.09
	F	04:19 ± 1:10					

Sleep architecture	Gender	m ± SD	Mann–Whitney's *U*	*p*	Effect size

TIB (hr)	M	7.46 ± 1.05	500	**0.002**	0.404
	F	8.34 ± 1.07			
SPT (hr)	M	7.30 ± 1.12	531	**0.004**	0.368
	F	8.17 ± 1.09			
TST (hr)	M	6.53 ± 1.15	444	**<0.001**	0.472
	F	7.52 ± 1.06			
SOL (min)	M	7.34 ± 2.47	808	0.769	0.038
	F	8.30 ± 4.45			
SE (TST/TIB%)	M	88.00 ± 10.13	600	**0.026**	0.285
	F	91.88 ± 4.15			
WASO (min)	M	37.51 ± 28.20	631	0.053	0.248
	F	25.11 ± 18.61			
WASO (%)	M	9.41 ± 7.01	572	**0.013**	0.313
	F	4.92 ± 3.52			

Sleep fragmentation	Gender	m ± SD	Mann–Whitney's *U*	*p*	Effect size

Number of awakenings ≥ 1 min	M	13.50 ± 6.68	748	0.393	0.110
	F	11.85 ± 5.42			
Mean duration of awakenings ≥ 1 min (min)	M	4.41 ± 3.48	745	0.383	0.113
	F	3.75 ± 1.54			
Frequency of awakenings ≥ 1 min/TSThr	M	2.03 ± 1.07	615	**0.037**	0.267
	F	1.51 ± 0.70			
Number of long awakenings (≥ 5 min)	M	3.39 ± 1.62	622	**0.042**	0.259
	F	2.66 ± 1.33			
Duration of longest awakening (min)	M	16.52 ± 10.80	808	0.770	0.038
	F	14.57 ± 7.21			
Frequency of long awakenings (≥ 5 min)/TSThr	M	0.56 ± 0.55	546	**0.006**	0.350
	F	0.34 ± 0.18			

Significant differences are in bold.

SE, sleep efficiency; SOL, sleep‐onset latency; SPT, sleep period time; TIB, time in bed; TST, total sleep time; WASO, wake after sleep onset.

### 3.4 Effects of DST

As displayed in Table 5, all sleep schedule variables appeared delayed in subjects who participated in the study after DST compared with those whose recordings were collected before that date. No other actigraphic variable showed between‐groups differences, except: sleep latency (before DST: 8.24 ± 4.07 min versus after DST: 6.40 ± 2.41 min, Mann–Whitney's *U* = 410, *p* = 0.038, effect size = 0.315), number of awakenings ≥ 1 min (before DST: 11.81 ± 5.95 versus after DST: 15.60 ± 5.89, Mann–Whitney's *U* = 373, *p* = 0.013, effect size = 0.376) and frequency of awakenings ≥ 1 min per TSThr (before DST: 1.67 ± 0.94 versus after DST: 2.16 ± 0.86, Mann–Whitney's *U* = 384, *p* = 0.019, effect size = 0.358).

**TABLE 5:** Differences in sleep schedules between subjects who participated in the study before DST and those who participated afterwards

Sleep schedules	Before/after DST	m ± SD	Student's *t*	*p*	Effect size	95%CI−	95%CI+
Bedtime (hr:min)	Before	00:21 ± 1:35	−2.03	**0.050**	0.400	−1.38	−0.00
	After	01:11 ± 1:32					
Wake time (hr:min)	Before	08:20 ± 1:20	−2.67	**0.011**	0.699	−1.22	−0.16
	After	09:16 ± 1:14					
Rise time (hr:min)	Before	08:28 ± 1:17	−2.73	**0.009**	0.714	−1.24	−018
	After	09:23 ± 1:12					
Sleep midpoint (hr:min)	Before	04:24 ± 1:19	−2.55	**0.016**	0.669	−1.19	−0.14
	After	05:15 ± 1:15					

Significant differences are in bold.

DST, Daylight Saving Time.

## 4 DISCUSSION

This is the first study to address objective and subjective sleep features during the third wave of the Covid‐19 pandemic. Actigraphic and PSQI data were collected from 82 healthy adults, during the lockdown imposed by the Italian government in March 2021 to confront the third wave of contagion.

Firstly, sleep schedules appear slightly delayed compared with what could be expected. In fact, we observed, through a longitudinal Italian survey, that sleep timing, initially delayed during the first pandemic wave (spring 2020), then linearly advanced when restrictions were lifted as well as through the second wave (autumn 2020; Conte, Cellini, et al., 2021). This trend suggested that sleep timing during the third wave would return to pre‐pandemic levels, i.e. bedtimes between 23:00 hours and midnight, and wake times generally not exceeding 08:00 hours (Cellini, Canale, et al., 2020; Cellini et al., 2021; Vitale et al., 2015). Instead, they were 00:33 hours and 08:33 hours, respectively, in our sample, which more closely approximates that observed during the first lockdown (Cellini, Canale, et al., 2020; Cellini et al., 2021; Ong et al., 2021). This appears surprising considering that the third lockdown was more similar to the second in terms of restrictions (which were looser relative to the first lockdown, with work routines partially recovered). However, we cannot exclude an effect of seasonal variations on sleep timing (Friborg, Bjorvatn, Amponsah, & Pallesen, 2012), which would be congruent with the similarities between the first and third lockdowns, or an effect of sample composition (differences in sleep timing between students, workers and unemployed individuals have been highlighted in several studies both before and during the pandemic; Cellini et al., 2020a, 2021).

Instead, TIB and sleep latency are coherent with the trend emerged in our longitudinal study (Conte, Cellini, et al., 2021), in which an increase of these measures during the first lockdown (confirmed by other pandemic studies; Cellini et al., 2021; Pépin et al., 2021) was followed by a return to baseline in the second. Indeed, the duration of TIB found here (8.09 hr) is very similar to that reported by Italian surveys before the pandemic (Cellini, Canale, et al., 2020; Cellini et al., 2021) as well as during the second pandemic wave (Conte, Cellini, et al., 2021). Also, sleep latency, which is < 10 min, approximates that observed by pre‐pandemic actigraphic studies (Cellini, Meneghini, et al., 2020; Tonetti, Erbacci, Fabbri, Martoni, & Natale, 2013), and is within the 15‐min limit recommended by the NSF among “good sleep quality” features (Ohayon et al., 2016).

Concerning sleep amount, our participants displayed almost 8 hr of SPT and 7.22 hr of TST. These data are not easily comparable to self‐report literature, considering that sleep duration is often underestimated (Jackson, Patel, Jackson, Lutsey, & Redline, 2018). As for objective data, although the few studies from the first wave were consistent on finding increased sleep duration with the lockdowns (Ong et al., 2021; Pépin et al., 2021; Sañudo et al., 2020), the total amount of sleep during the lockdown varies among studies from about 6.5 hr (Ong et al., 2021; Pépin et al., 2021) to more than 8 hr (Sañudo et al., 2020; Wang et al., 2021). Also, none of these studies provided operational definitions of their sleep duration measure, allowing to distinguish between TST and SPT. Nevertheless, our results on both measures suggest sufficient sleep duration in our sample according to the NSF’s 7–9 hr recommended range (Hirshkowitz et al., 2015), which confirms that sleep amount was relatively spared by the negative impact of the pandemic (Cellini et al., 2021; Ong et al., 2021; Wang et al., 2021). However, note that actigraphy tends to overestimate sleep and underestimate wakefulness (Goldstone, Baker, & de Zambotti, 2018).

Our results on subjective sleep quality confirm the trend observed in longitudinal surveys, which showed that its impairment remained high during the second lockdown (Conte, Cellini, et al., 2021; Salfi et al., 2021). Indeed, the average PSQI global score in our sample, though lower than that reported during the second wave (Conte, Cellini, et al., 2021; Salfi et al., 2021), is higher than the cut‐off for poor sleep (Buysse et al., 1989), and more than half of our participants are classified as poor sleepers.

These findings apparently contradict those on objective sleep quality. In fact, in line with objective sleep studies from the first wave (Ong et al., 2021; Pépin et al., 2021; Sañudo et al., 2020; Wang et al., 2021),we did not find a relevant impairment of classical sleep quality measures. As in Ong et al. (2021) and Pépin et al. (2021), SE is within the recommended range (i.e. above 85%; Ohayon et al., 2016). WASO time (31 min) shows liminal values, being slightly higher than that recommended by the NSF (≤ 20 min; Ohayon et al., 2016), but falls within the normal range when considering its percentage over SPT (Berger et al., 2005). However, more specific sleep continuity measures reveal the presence of frankly disrupted sleep. Indeed, the number of long awakenings (≥ 5 min) exceeds the limit considered as indicative of good sleep in adults (0–1 per night; Ohayon et al., 2016). Also, the total frequency of awakenings lasting ≥ 1 min is considerably higher than that found in good sleepers (Conte, Cerasuolo, et al., 2021). Considering previous literature pointing to number of awakenings as a main determinant of perceived sleep quality (Conte, Cerasuolo, et al., 2021; Della Monica et al., 2018), the relevant sleep fragmentation observed in our participants may also explain their poor sleep perception.

Along the same line of reasoning, it may be hypothesized that the significant impairments of subjective sleep quality widely reported during the first pandemic wave (Casagrande et al., 2020; Cellini, Canale, et al., 2020; Cellini et al., 2021) could have relied on the presence of subtle objective sleep quality disruptions. These would have gone undetected by objective sleep assessments, performed during the same period, which did not include fragmentation indices (Ong et al., 2021; Pépin et al., 2021; Sañudo et al., 2020; Wang et al., 2021). To this regard, it is worth noting that, in our previous longitudinal study, self‐reported number of awakenings and their average duration showed a profile of changes across the pandemic waves parallel to that of general subjective sleep quality (PSQI global score), i.e. a significant worsening during the first lockdown, followed by a return to baseline during the period with no restrictions and a renewed worsening during the second lockdown (Conte, Cellini, et al., 2021).

Interestingly, gender differences emerged for most objective sleep variables. Women showed earlier sleep schedules, stayed in bed and slept longer, displayed higher SE, lower WASO% and lower sleep fragmentation. In other words, despite the absence of gender differences in subjective sleep quality, women slept much better than men (in line with findings from a pre‐pandemic actigraphic study on university students; Cellini, Meneghini, et al., 2020). Actually, although the differences were non‐significant, women's PSQI scores were even higher than men's (both their global score and all but two sub‐scores), in line with numerous studies pointing to female gender as a risk factor for greater worsening of subjective sleep quality with the pandemic (Casagrande et al., 2020; Cellini et al., 2021). This striking subjective/objective dissociation in women is not surprising in light of pre‐pandemic literature on sleep quality in the general population. Indeed, as highlighted in Mong and Cusmano (2016), while women display better PSG‐defined sleep quality than men (Ohayon, Carskadon, Guilleminault, & Vitiello, 2004), they report disrupted and insufficient sleep more frequently than men in a wide range of subjective studies (Groeger, Zijlstra, & Dijk, 2004). Therefore, our findings show that this general trend is still present during the pandemic, and possibly is even exacerbated by it.

Finally, our analysis of possible differences between actigraphic recordings collected before and after the introduction of DST revealed that sleep schedules were delayed by about an hour in subjects whose recordings were collected after the time change. Moreover, sleep latency was reduced in the latter group, possibly indicating increased sleepiness, whereas sleep fragmentation, as indexed by the number and frequency of brief awakenings, was increased. These findings are coherent with literature on the effects of spring transitions into DST (Tonetti et al., 2013), and suggest that the deterioration of the sleep/wake cycle linked to the third wave of the pandemic emergency may have been worsened by the concomitant transition into DST.

Our limited sample size and limited number of recording nights (compared with the minimum 5 nights recommended by some authors; Aili, Åström‐Paulsson, Stoetzer, Svartengren, & Hillert, 2017) impose caution in interpreting our results. However, these caveats should be appraised in light of the numerous limitations imposed by the pandemic emergency. First, the unpredictability of changes in restrictions: since November 2020, the Italian government started imposing lockdowns that were graded by severity according to regional case rates, and changes in restrictions were announced with just a few days of notice. Therefore, the planning phase of the research had to be conducted within this very brief time span. Indeed, our choice of a limited number of recording nights was specifically driven by this condition (i.e. once initiated, the end of the lockdown could not be predicted), balanced by the need to enroll a sufficiently numerous sample. Moreover, general fear of contagion significantly slowed down the recruitment process, despite the fact that procedures were conducted in strict accordance with health guidelines.

On the other hand, although our choice of using objective measurements unavoidably narrowed sample size, this methodology also represents the main strength of this research. In fact, unsurprisingly, very few studies have performed objective sleep assessments in previous phases of the pandemic. In addition, our in‐depth evaluation of sleep fragmentation provides the first evidence, during the pandemic, of subtle sleep disruptions that could be masked by the appearance of general good sleep quality according to classical parameters (such as SE). Indeed, it has been repeatedly proposed that more fine‐grained analyses of sleep could be more adequate to evaluate its objective quality (Klerman et al., 2013; Norman, Scott, Ayappa, Walsleben, & Rapoport, 2006).

In conclusion, our study contributes to describe the temporal profile of sleep across the different phases of this prolonged pandemic emergency. We highlight that, during the third wave, sleep is characterized by significant objective sleep fragmentation in the face of adequate sleep duration, suggesting a greater impoverishment of sleep quality than what could be expected from objective sleep studies conducted during the first wave. Taken together with sleep data on previous phases of the pandemic, our findings show that the detrimental effects on sleep determined by the initial outbreak of the pandemic, with the abrupt implementation of strict confinement procedures, have not abated across the subsequent waves of contagion and related confinement periods. In this perspective, the recurrent and unpredictable periods of reinforced restrictions (and related social and financial costs), occurring over the course of the global health crisis, may be viewed as a form of “acute‐on‐chronic stress” (Gabrielli & Lund, 2020), with profound effects on sleep and well‐being, which should be addressed by researchers, clinicians and politicians worldwide.

## CONFLICT OF INTEREST

The authors declare no conflicts of interest, and no personal or financial involvement with any organization having financial interest in the subject matter of the paper.

## AUTHOR CONTRIBUTIONS

All authors contributed in a meaningful way to this manuscript. F.C., P.S., F.G., G.B. and G.F.: conceptualization; F.C., O.D. and G.F.: methodology; O.D., P.D. and A.L.: formal analysis; O.D., M.R., T.P., A.L., S.M. and D.M.: investigation; F.C., O.D. and M.R.: writing—original draft preparation; F.C., O.D. and G.F.: writing—review and editing; F.C., O.D. and G.F.: visualization; P.S., F.G., G.B. and G.F.: supervision; P.S., F.G., G.B. and G.F.: project administration. All authors have read and agreed to the submitted version of the manuscript.

## Data Availability

The data that support the findings of this study are available from the corresponding author upon reasonable request.
